# The apoptotic mechanisms of MT-6, a mitotic arrest inducer, in human ovarian cancer cells

**DOI:** 10.1038/srep46149

**Published:** 2017-04-07

**Authors:** Mei-Chuan Chen, Yi-Chiu Kuo, Chia-Ming Hsu, Yi-Lin Chen, Chien-Chang Shen, Che-Ming Teng, Shiow-Lin Pan

**Affiliations:** 1 Ph.D. Program for the Clinical Drug Discovery from Botanical Herbs, College of Pharmacy, Taipei Medical University, Taipei, Taiwan; 2 Pharmacological Institute, College of Medicine, National Taiwan University, Taipei, Taiwan; 3 Ph.D. Program for Cancer Biology and Drug Discovery, College of Medical Science and Technology, Taipei Medical University, Taipei, Taiwan; 4 National Research Institute of Chinese Medicine, Ministry of Health and Welfare, Taipei, Taiwan

## Abstract

Patients with ovarian cancer are typically diagnosed at an advanced stage, resulting in poor prognosis since there are currently no effective early-detection screening tests for women at average-risk for ovarian cancer. Here, we investigated the effects of MT-6, a derivative of moscatilin, in ovarian cancer cells. Our investigation showed that MT-6 inhibited the proliferation and viability of ovarian cancer cells with submicromolar IC_50_ values. MT-6–treated SKOV3 cells showed significant cell cycle arrest at G2/M phase, followed by an increase in the proportion of cells in a sub-G1 phase. In addition, MT-6 induced a concentration-dependent increase in mitotic markers, mitotic kinases, cell cycle regulators of G2/M transition, and apoptosis-related markers in ovarian cancer cells. MT-6 treatment also induced mitochondrial membrane potential loss, JNK activation, and DR5 expression. Cotreatment of cells with the JNK inhibitor SP600125 considerably attenuated MT-6–induced apoptosis, mitochondria membrane potential loss, DR5 upregulation, and suppression of cell viability. MT-6 also inhibited tumor growth in an SKOV3 xenograft model without significant body weight loss. Together, our findings suggest that MT-6 is a potent anticancer agent with tumor-suppressive activity *in vitro* and *in vivo* that could be further investigated for ovarian cancer therapy in the future.

Among malignant gynecological tumors, patients with ovarian cancer have a high mortality rate owing to late stage diagnosis^1^. In addition to debulking surgery, the standard treatment for ovarian cancer is platinum-based chemotherapy in combination with taxane cytotoxic drugs, but a majority of these patients ultimately relapse within 2 years^2^. Therefore, prolonged courses of chemotherapy or better therapeutic options need to be continuously investigated. Antimitotic agents, which produce significant cytotoxicity, have been used effectively in the clinic for decades in patients with a variety of malignancies, including breast cancer, ovarian cancer, and lung cancer^3^,^4^. Although current trends of drug development for cancer treatment emphasize target-oriented approaches to enhance specificity so as to reduce unwanted side effects, novel antimitotic drugs still retain significant clinical value and have yielded promising outcomes^5^,^6^,^7^.

During the cell cycle, progression from G2 to M phase requires activation of the Cdk1/cyclin B1 complex, which is controlled by phosphorylation at different sites of Cdk1^8^,^9^. Antimitotic agents usually target microtubule dynamics and cell-cycle regulatory proteins, whose main function is to properly coordinate cell division in mammalian cells. Consequently, antimitotic drugs cause cell cycle dysregulation (mitotic arrest) followed by aberrant division and cell death^10^. Apoptosis, the best-known form of programmed cell death, mainly involves activation of a cascade of caspase that is triggered by the extrinsic (death receptor) or intrinsic (mitochondrial) apoptotic pathways and leads to characteristic biochemical and morphological changes^11^,^12^. The intrinsic apoptotic pathway is characterized by mitochondrial outer membrane permeabilization (MOMP) and is regulated by functionally distinct members of the BCL-2 family of proteins through interactions between and among anti- and pro-apoptotic members^13^. On the other hand, the extrinsic apoptotic pathway is initiated by members of the tumor necrosis factor (TNF) receptor superfamily and spreads to other apoptotic signal transduction cascades^14^. Death receptor 5 (DR5/TRAILR-2) is one of five known members of the TRAIL (tumor necrosis factor apoptosis-inducing ligand) receptor family, also known as type II membrane bound TNF family ligand receptors^15^. Activation of DR5 induces formation of death-inducing signaling complexes (DISC), which in turn promote caspase 8/10 oligomerization and activation, leading to subsequent cleavage and release of the active initiator caspase^16^. It has further been reported that loss of DR5 function in gastric carcinomas and head-and-neck cancer may cause loss of growth-suppressive function^17^,^18^, suggesting that DR5 exhibits cell-killing activity, and thus is a candidate tumor-regulator protein.

Numerous compounds derived from natural products have been shown to confer significant antitumor activities and may have the potential to circumvent drug resistance^19^. Moscatilin (MT), a bibenzyl component derived from the India orchid *Dendrobium moscatum* and the stem of *Dendrobium loddigesii,* has been reported to exert cytotoxicity toward malignant cells and inhibit platelet aggregation^20^,^21^. MT-6, belonging to a series of MT-derivatives, has shown potency in numerous cancer cell lines. Here, we show for the first time that MT-6, a potent mitotic inhibitor, induces apoptotic cell death through activation of c-Jun N-terminal kinase (JNK) and induction of DR5 in SKOV3 ovarian cancer cells. These findings may provide a new strategy for ovarian cancer treatment, either alone or in combination with other therapeutic agents.

## Materials and Methods

### Cell lines and reagents

Non-small cell lung cancer cells (A549), colorectal cancer cells (HT29), ovarian cancer cells (A2780, OVCAR3 and SKOV3), Hepatocellular carcinoma cells (Hep3B), breast cancer cells (MDA-MB0231) and uroepithelium cells (SV-HUC-1) were obtained from the American Type Culture Collection (ATCC) (Manassas, VA, USA). Cells were maintained in 10% fetal bovine serum (FBS)-supplemented RPMI 1640 or F12K medium (GIBCO, Grand Island, NY, USA) and 1% penicillin-streptomycin (GIBCO) at 37 °C in a humidified incubator containing 5% CO_2_. MT-6, 5-(3-fluro-4-methoxyphenethyl)-1,2,3-trimethoxybenzene, was obtained from Dr. Chien-Chang Shen (National Research Institute of Chinese Medicine, Ministry of Health and Welfare, Taipei, Taiwan). Antibodies against various proteins were obtained from the following sources: PARP (Poly-ADP-ribose polymerase), cyclin B1, Bcl-2, Mcl-1, Bcl-xL, and anti-mouse and anti-rabbit IgGs were obtained from Santa Cruz Biotechnology Inc. (Dallas, TX, USA). Caspase 8, caspase 9, caspase 7, caspase 10, phospho-cdc2 (T161), phospho-cdc2 (Y15), phospho-PLK (T210), phospho-Aurora B (T232), Bcl-2 (Ser70), phospho-Akt (Ser473), phospho-p38, Akt, phospho-p44/42 MAPK (1/2 Erk) (Thr202/Tyr204), and phospho-JNK were obtained from Cell signaling (Danvers, MA, USA). Beta-actin, MPM-2, and phospho-histone H3 were from Millipore (Billerica, MA, USA). Caspase 3 and was obtained from Novus (Littleton, CO, USA). SP600125 and Rhodamine 123 were obtained from Sigma (St. Louis, MO, USA). FITC-labeled DR5 antibody was purchased from ThermoFisher Scientific (Waltham, MA, USA).

### Cell viability assay

Cells were seeded into 96-well plates and cultured overnight followed by the exposure to various concentrations of indicated drugs for 24 h or 48 h. Cell viability was assayed by the 3-(4,5-dimethylthiazol-2-yl)-2,5-diphenyltetrazolium bromide assay as described previously^20^. Growth inhibition was expressed as the percentage of surviving cells in drug-treated versus DMSO-treated control cells (which was considered as 100% viability).

### SRB (sulforhodamine B) assay

Cells were seeded into 96-well plates and cultured overnight followed by the exposure to various concentrations of indicated drugs for 48 h. Cells were then fixed *in situ* with 10% trichloroacetic acid (TCA) to represent a measurement of the cell population at the time of drug addition (T0). After an additional 48 h incubation with or without MT-6 in medium with 5% FBS, the assay was terminated by 10% TCA. SRB dye at 0.4% (w/v) in 1% acetic acid was added to stain the cells. Unbound dye was removed by washing with 1% acetic acid twice and the plates were air dried. Bound dye was subsequently solubilized with 10 mM trizma base, and the absorbance was read at a wavelength of 515 nm. Growth inhibition of 50% (GI_50_) is calculated as described previously^22^.

### FACScan flow cytometric analysis

Cells were seeded in 6-well plates (2.5–3 × 10^5^/well) and treated with DMSO or MT-6 at various concentrations for indicated times. Cells were washed with phosphate-buffered saline, fixed in ice-cold 70% ethanol at −20 °C overnight, and stained with propidium iodide (80 μg/ml) containing Triton X-100 (0.1%, v/v) and RNase A (100 μg/ml) in phosphate-buffered saline. DNA content was analyzed with the FACScan and CellQuest software (Becton Dickinson, Mountain View, CA, USA). For determination of DR5 expression, cells were incubated in PBS containing FITC-conjugated DR5 antibodies against the extracellular domain of DR5 for 30 minutes at room temperature. For determination of mitochondria membrane potential, cells were incubated with 10 μM rhodamine 123 at 37 °C for 30 minutes before the end of incubation. Cells were harvested and rhodamine 123 accumulation was determined by flow cytometric analysis.

### Caspase activity assays

Caspase colorimetric activity assay were done as described by the manufacturer. Briefly, reaction mixtures were assembled as follows: 150 to 200 μg protein (whole-cell lysates from drug-treated SKOV3 cells), 50 μL of 2 × reaction buffer (containing 10 mmol/L DTT), and 5 μL peptide substrate (LEHD- p NA for caspase-9 assays, IETD-p NA for caspase-8 assays, or DEVD-p NA for caspase-3 assays). Reactions were incubated at 37 °C for 1 h and read at 405 nm by an ELISA reader.

### Transient transfection

Cells were seeded into 6-well plate and incubated overnight for attachment. JNK siRNA was purchased from Dharmacon (Lafayette, CO, USA) and the cells were transfected with Lipofectamine RNAiMAX transfection reagent (Invitrogen) according to the manufacturer’s instruction. After 24 h, the cells were exposed to MT-6 for another 24 or 48 h, and total cell lysates were collected and subjected to western blot analysis.

### Immunoblotting

Cells were seeded in 6-cm dishes and allowed to attach for overnight. The cells were treated with MT-6 or combination with SP600125 with indicated concentrations for indicated times. After the indicated exposure time, cells were lysed and the immunoblotting was performed as previous described^23^. Briefly, cell lysates were extracted by lysis buffer and proteins were quanitified by BCA Protein Assay Kit (Thermo Scientific, Rockford, IL, USA). Equal amount of protein was resolved by 8~15% SDS-PAGE, then transferred onto PVDF membrane. After blocking with TBST containing 5% nonfat milk for 1 h, the membrane was incubated with appropriate primary antibody at 4 °C overnight. After washing thrice with TBST for total 30 min, followed by goat anti-mouse or anti-rabbit IgG-HRP conjugates for 1 h at room temperature, and washed thrice with TBST for total of 30 min. The immnunoblots were visualized by enhanced chemiluminescence detection kit (Amersham, England).

### Confocal microscopy

After treatment with MT-6, the cells were washed twice with PBS and fixed with methanol for 20 min at −20 °C. Then, the slides were blocked with bovine serum albumin (1%, w/v) containing Triton X-100 (0.1%, v/v) in PBS at 37 °C for 1 h at room temperature. Then cells were washed twice with PBS and incubated with anti-β-tubulin antibody (1:500 dilution) at 37 °C for 1 h. After the incubation, cells were washed twice with PBS and stained with FITC-conjugated anti-mouse IgG antibody (1:400 dilution) plus DAPI (1 μg/ml) in PBS for 45–60 min at room temperature in the dark. The slides were washed twice with PBS and imaged with Leica TCS SP2 Spectral Confocal System.

### *In vitro* tubulin polymerization assay

Tubulin proteins ( > 99% purity, CytoDYNA-MIX ScreenTM3 kit, Cytoskeleton Inc., Denver, CO) were suspended (300 μg) with 100 μl of G-PEM buffer (80 mM PIPES, 2 mM MgCl_2_, 0.5 mM EGTA, 1.0 mM GTP, pH6.9) plus 5% glycerol in the absence or presence of indicated compounds at 4 °C. The sample mixture was transferred to the prewarmed 96-well plate, and the polymerization of tubulin was measured by the change in absorbance at 340 nm every 1 min for 30 min (SpectraMAX Plus; Molecular Devices, Inc., Sunnyvale, CA) at 37 °C.

### SKOV3 xenograft models

Athymic nu/nu female mice were obtained from the National Laboratory Animal Center (Republic of China, Taiwan) and maintained in pathogen free conditions. Six mice per group were used in the xenograft studies. Mice were implanted subcutaneously with 1 × 10^6^ SKOV3 cells per mouse. When the tumors reached the average volume of 100 mm^3^, the mice were divided into four groups (n = 6) and the treatments were initiated. Vehicle (Cremophor EL/ethanol, 1:1; 0.2 mL/mouse) or MT-6 at doses of 5 and 10 mg/kg were administered i.p. once daily. The length (L) and width (W) of the tumor were measured every 3 to 4 days, and the tumor volume was calculated as LW^2^ / 2. Animal experiments were performed in accordance with relevant guidelines and regulations followed ethical standards, and protocols has been reviewed and approved by Animal Use and Management Committee of Taipei Medical University (LAC-2013–0139).

### Statistics and data analysis

Each experiment was performed at least three times, and presentative data are shown. Data in bar graph are given as the means ± S.E.M. Means were checked for statistical difference using the t-test and *P*-values less than 0.05 were considered significant (*P < 0.05, **P < 0.01, ***P < 0.001).

## Results

### Cell-growth inhibitory effects of MT and its derivatives in different cancer cell lines

**Figure 1:**
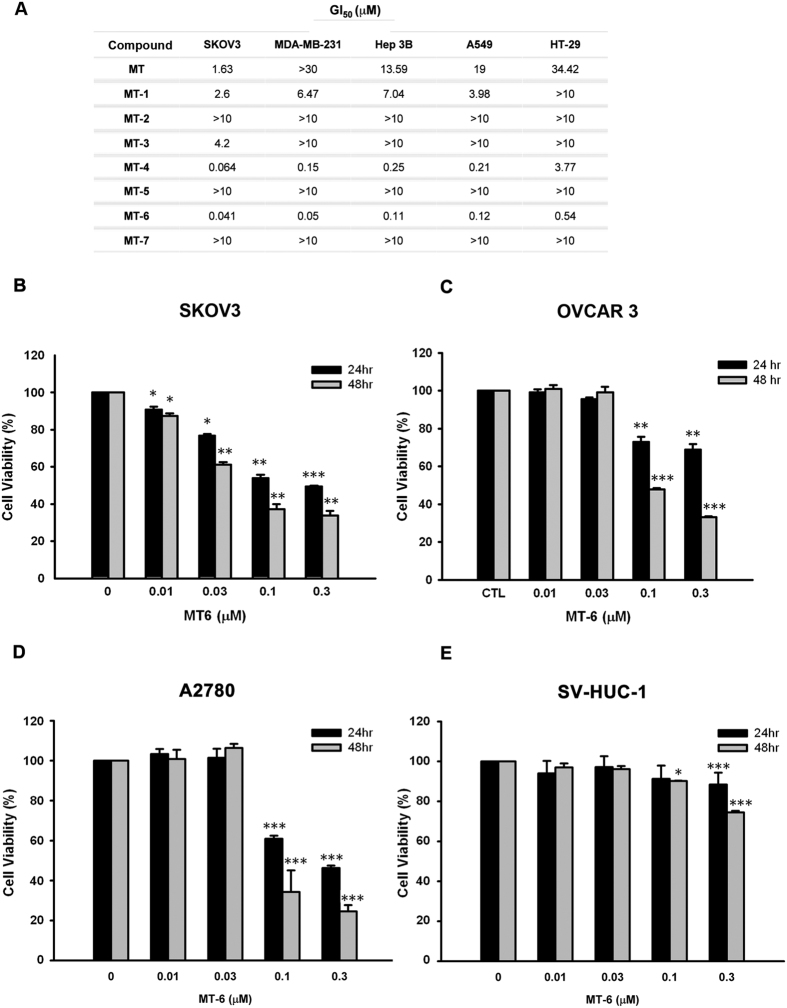
MT-6 induces apoptosis in human ovarian cancer cells. (**A**) GI_50_ values for MT and its derivatives in various cancer cell lines. Cells were treated with different concentrations of the indicated drugs for 48 h, and cell-growth inhibition was assessed by SRB assays. (**B–D**) Concentration-dependent effects of MT-6 on the viability of ovarian cancer cells. Cells were treated with MT-6 at the indicated concentrations for 24 or 48 h, and the viability of SKOV3 (**B**), OVCAR3 (**C**) and A2789 (**D**) cells was analyzed by MTT assay. (**E**) Viability of normal uroepithelium cells (SV-HUC-1) following treatment with MT-6. Cells were incubated with or without the indicated concentrations of MT-6 for 24 or 48 h, and cell growth was evaluated by MTT assay. Data are presented as means ± S.E.M. of three independent experiments (*P < 0.05, **P < 0.01 and ***P < 0.001 compared with non-treated cells).

To determine the *in vitro* antitumor activity of the compound MT and its derivatives, we performed sulforhodamine B (SRB) assays in various cancer cell lines treated with different concentrations of drugs. These assays revealed that MT-6 most potently inhibited the proliferation of the tested tumor cells, with 50% growth-inhibitory (GI_50_) values in the submicromolar range (Fig. 1A). We further examined cell viability in SKOV3 (Fig. 1B), OVCAR3 (Fig. 1C), and A2780 (Fig. 1D) ovarian cancer cells, as well as normal uroepithelial cells (SV-HUC-1) (Fig. 1E) following treatment with MT-6. These experiments revealed significant and concentration-dependent inhibitory effects of MT-6 in cancer cells without apparent cytotoxicity toward SV-HUC-1 cells, indicating that MT-6 has good selectivity for tumor cells.

### MT-6 induces mitotic arrest followed by apoptosis in ovarian cancer cells

**Figure 2:**
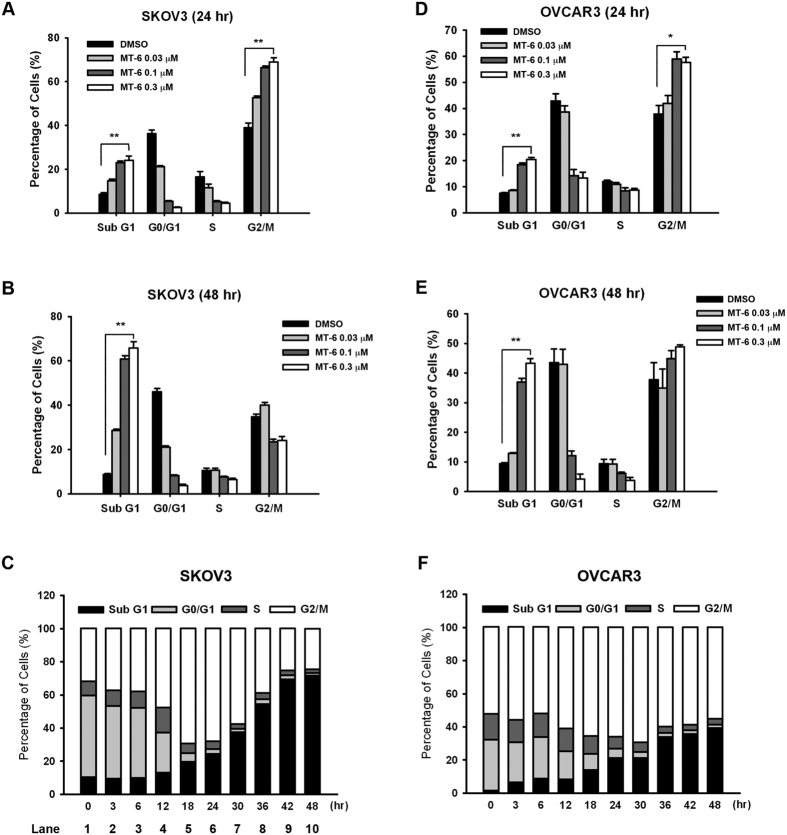
Effects of MT-6 on cell cycle distribution in ovarian cancer cells. SKOV3 (**A,B**) or OVCAR3 (**D,E**) cells were treated with different concentrations (0.01–0.3 μM) of MT-6. After incubation for 24 h (**A** and **D**) or 48 h (**B** and **E**), the cell cycle was analyzed by flow cytometry after propidium iodide staining. Quantitative data (**C** and **F**) are based on flow cytometry histograms, and are presented as means ± S.E.M. of at least three independent experiments that yielded similar results (*P < 0.05 and **P < 0.01 compared with non-treated cells).

To investigate the underlying mechanism of MT-6–induced cell-growth repression, we examined the effects of MT-6 on cell-cycle progression using flow cytometry. Treatment of SKOV3 cells with MT-6 for 24 h induced a significant concentration-dependent accumulation of cells in G2/M phase (Fig. 2A), whereas treatment for 48 h caused a clear increase in a subG1 cell population (Fig. 2B). As shown in Fig. 2C, cells began to accumulate in G2/M within 3 h of initiating treatment (lane 2), with a maximum occurring after 18 h treatment (lane 5); thereafter (18–24 h), subG1 phase cells started to accumulate (lane 5 to lane 10). Similar effects were also observed in OVCAR3 cells (Fig. 2D–F). Together, our data suggest that MT-6 induces G2/M-phase arrest and apoptosis in ovarian cancer cells. Our results showed that SKOV3 cells were more sensitive to MT-6 than OVCAR3 cells, and were therefore chosen for all subsequent experiments.

### Effects of MT-6 on G2/M phase of the cell cycle and apoptotic regulatory proteins

**Figure 3:**
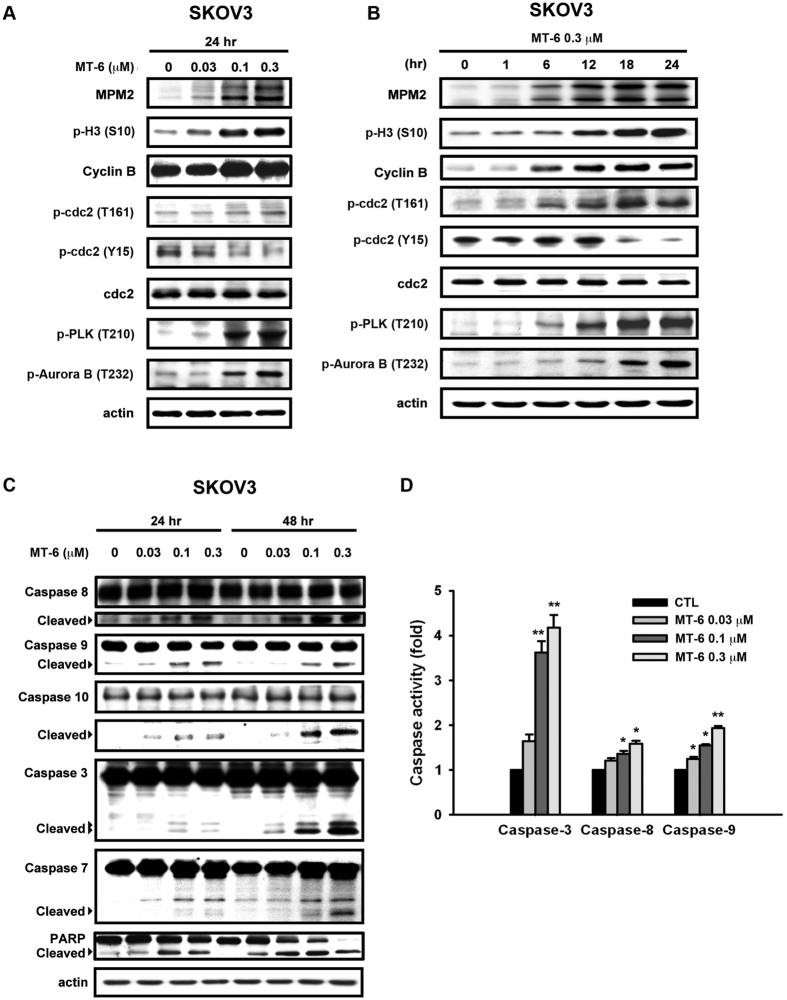
Effects of MT-6 on markers of mitotic arrest and apoptosis in ovarian cancer cells. (**A**,**B**) MT-6 induces increases in mitotic markers and cell cycle regulators at the G2/M transition in a concentration- (**A**) and time- (**B**) dependent manner. (**C**,**D**) Effects of MT-6 on apoptosis in SKOV3 cells. MT-6 increases levels of the cleaved (activated) forms (**C**) and activity (**D**) of caspase-3, -8, and -9. Cells were treated with the indicated concentrations of MT-6 for the indicated times, and whole-cell extracts were analyzed by Western blotting.

We noted consistent increases in the expression levels of the general mitotic markers, MPM-2, cyclin B, and phosphorylated histone H3 (Ser10) following MT-6 treatment (Fig. 3A), suggesting that the accumulation of cells in G2/M was attributable to mitotic arrest. In addition, we also observed concentration- and time-dependent phosphorylation of the activating Thr161 residue of Cdk1 and dephosphorylation of the inhibitory Tyr15 residue of Cdk1, suggesting that MT-6 activates the Cdk1/cyclin B1 complex in SKOV3 cells (Fig. 3A and B). Since the expression levels of two mitotic kinases, PLK1 (Polo-like kinase-1) and Aurora kinase B, play important roles in mitotic progression and regulation of cell division^24^,^25^,^26^, we further investigated the expression levels of these two activated kinases. These experiments revealed that MT-6 treatment induced a concentration- and time-dependent accumulation of both phosphorylated PLK1 (p-PLK1) and p-Aurora B kinase (Fig. 3A and B). Moreover, apoptotic cells containing activated (cleaved) caspase-3, -7, -8, -9 and -10, and poly-(ADP-ribose) polymerase (PARP) were detected after treating with MT-6 for 24 h (Fig. 3C and D). MT-6-mediated mitotic arrest and apoptosis were also observed in OVCAR3 and A2780 cells, excluding the possibility of cell line-specific effects (Supplementary Fig. S1). Together, these results indicate that MT-6 induces G2/M-phase arrest and apoptotic cell death in SKOV3 cells.

### Ehffects of MT-6 on mitochondria membrane potential and Bcl-2 family proteins

**Figure 4:**
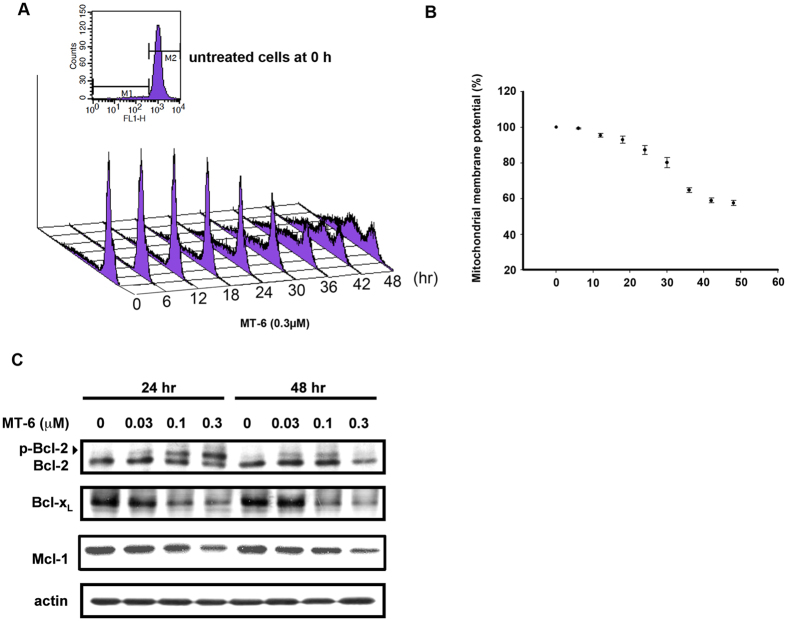
Effects of MT-6 on Δψ_m_ and Bcl-2 family proteins in ovarian cancer cells. (**A**,**B**) MT-6 induces Δψ_m_ loss in a time-dependent manner. Cells were treated with 0.3 μM MT-6 for different durations, and Δψ_m_ was (**A**) measured by flow cytometry following rhodamine 123 staining (10 μM) and (**B**) expressed as percentage loss compared with control cells. (**C**) MT-6 downregulates pro-survival Bcl-2 family proteins. SKOV3 cells were treated with different concentrations of MT-6. After incubation for 24 or 48 h, whole-cell lysates were analyzed by Western blotting. Similar results were obtained in at least three independent experiments.

Rhodamine 123 (Rh123), a membrane-permeable cationic fluorescent dye, shows reduced staining intensity in cells with mitochondrial damage, an early feature of apoptotic cells, and is used as a probe to detect changes in mitochondrial membrane potential (Δψ_m_)^27^,^28^. To investigate whether MT-6–induced apoptosis involves mitochondrial dysfunction, we measured Δψ_m_ using flow cytometry after staining of drug-treated cells with Rh123. As shown in Fig. 4A and B, MT-6 induced a significant, time-dependent increase in the population of cells with relatively low Rh123 staining (left-gated M1 phase), suggesting that MT-6 leads to a loss of Δψ_m_ and mitochondrial damage in SKOV3 cells. Moreover, it has been reported that members of the Bcl-2 family have a major role in the regulation of mitochondrial outer membrane permeabilization (MOMP), and increased expression of Bcl-2, Bcl-_XL_ and Mcl-1 has been reported in human solid tumors^13^. Our data also indicate that MT-6 reduced expression levels of the pro-survival proteins, Bcl-2, Mcl-1, and Bcl-_XL_ (Fig. 4C). Collectively, these results suggest that MT-6 has the capacity to interfere with Δψ_m_ and downregulate anti-apoptotic proteins in SKOV3 ovarian cancer cells.

### MT-6 upregulates protein expression levels of DR5 in SKOV3 cells

**Figure 5:**
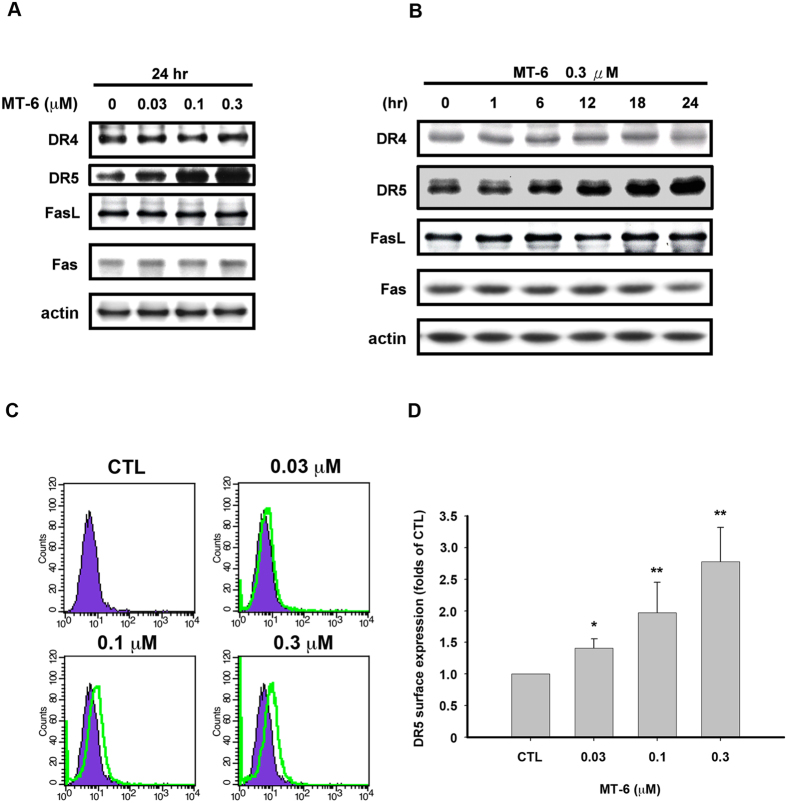
Effects of MT-6 on expression levels of death receptor proteins in ovarian cancer cells. (**A,B**) MT-6 increases DR5 expression levels in SKOV3 cells in a concentration- (**A**) and time- (**B**) dependent manner. SKOV3 cells were treated with the indicated concentrations of MT-6 for the indicated times, then whole-cell lysates were analyzed by Western blotting. (**C**,**D**) MT-6 induces DR5 expression on the cell surface, as measured by detection of a FITC-labeled DR5 antibody by flow cytometry. SKOV3 cells were treated with different concentrations of MT-6 for 24 h, and DR5 expression on cell surface was detected by flow cytometry as described in Materials and Methods. Similar results were obtained in at least three independent experiments. (* *P < *0.05 and ** *P < *0.01 compared with non-treated cells).

Our data shown in Fig. 3C suggest that MT-6 activates cleavage of caspase-8 and -9, representing extrinsic (death receptor) and intrinsic (mitochondria) pathways of apoptosis, respectively. Previous studies have indicated that TRAIL-induced cell death signaling is related to both extrinsic and intrinsic apoptotic pathways, and may be a promising therapeutic pathway for targeting by single agents or combined treatment with conventional chemotherapies^29^,^30^. To delineate the mechanism of MT-6–induced cell death, we further examined the levels of death receptors for pro-apoptotic signaling, such as transmembrane death receptors belonging to the tumor necrosis factor receptor (TNFR) superfamily. As shown in Fig. 5A and B, MT-6 upregulated the level of DR5 (TRAIL receptor 2/TNFRSF10) in cells without appreciably affecting DR4, Fas, or FasL. Using flow cytometry and a FITC-labeled DR5 antibody to further analyze the amount of DR5 on the cell surface of SKOV3 cells, we found that MT-6 induced a concentration-dependent increase in DR5 protein (Fig. 5C and D). These results suggest that DR5 may play a role in MT-6–induced cytotoxicity in ovarian cancer cells.

### The role of JNK in MT-6–induced cytotoxicity in ovarian cancer cells

**Figure 6:**
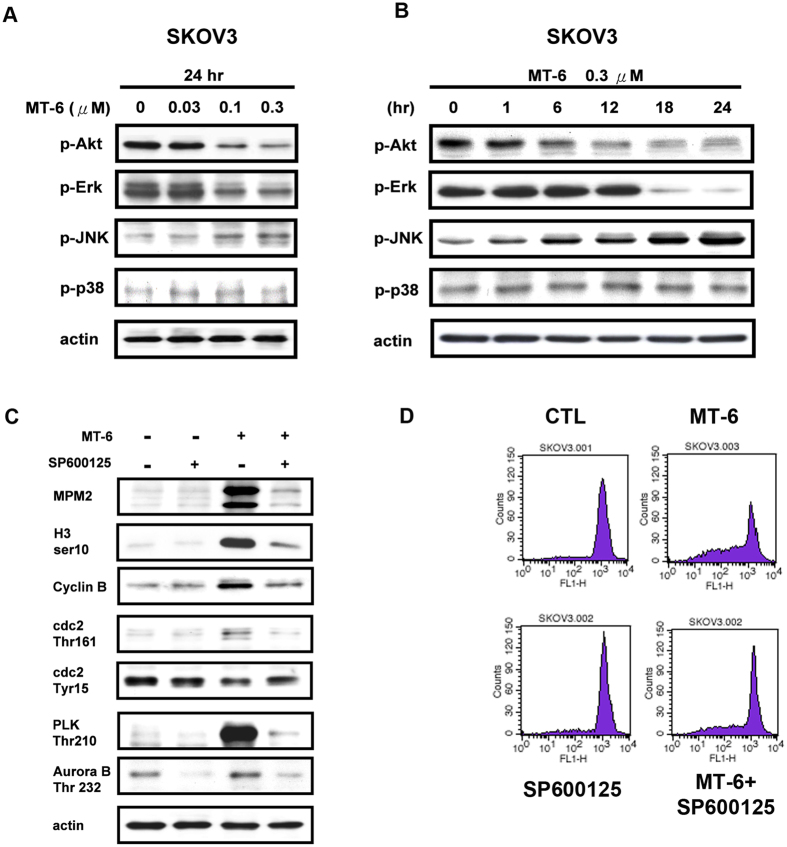
MT-6–induced Δψ_m_ loss and mitotic arrest are JNK dependent. (**A,B**) MT-6 upregulates p-JNK levels in a concentration- (**A**) and time- (**B**) dependent manner. Effects of MT-6 on survival signaling were investigated by Western blot analysis. (**C**) MT-6–induced mitotic arrest was reversed by cotreatment with the JNK inhibitor SP600125. SKOV3 cells were treated with the indicated concentrations of MT-6 for the indicated times, with or without SP600125 cotreatment, then whole-cell lysates were analyzed by Western blotting. (**D**) Cotreatment with the JNK inhibitor SP600125 substantially attenuates MT-6–induced Δψ_m_ loss. Cells were treated with 0.3 μM MT-6 for 24 h, then Δψ_m_ was measured by rhodamine 123 (10 μM) staining followed by flow cytometry analysis.

**Figure 7:**
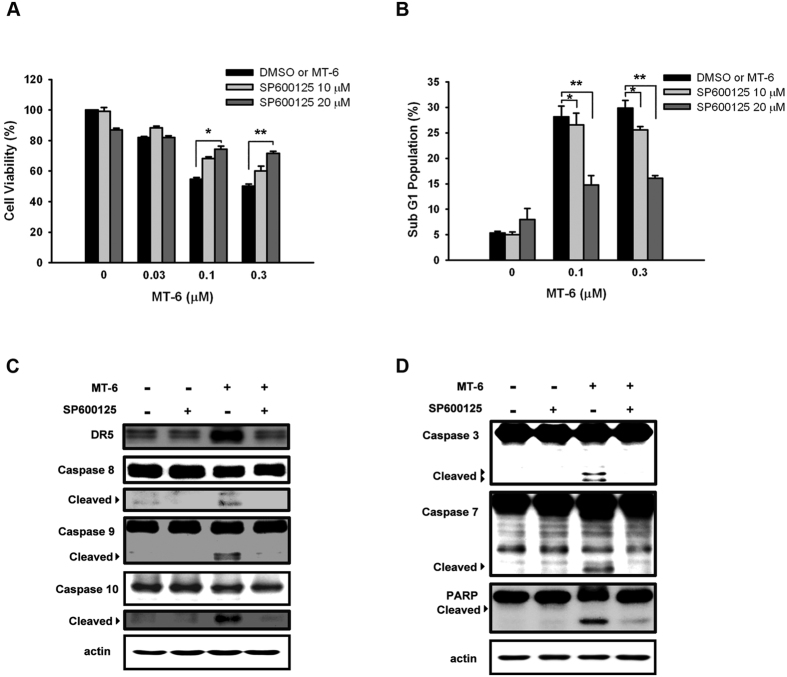
MT-6–induced apoptotic cell death is mediated by JNK activation. (**A**) JNK inhibition protects against MT-6–mediated inhibition of cell viability. (**B**) MT-6–induced accumulation of subG1 phase cells was attenuated by cotreatment with the JNK inhibitor SP600125. (**C**,**D**) Inhibition of JNK substantially attenuates MT-6–induced caspase activation and PARP cleavage in SKOV3 cells. SKOV3 cells were treated with the indicated concentrations of MT-6 for 48 h, with or without cotreatment with the JNK inhibitor SP600125, then whole-cell lysates were analyzed by Western blotting.

To further investigate the mechanism of MT-6–induced cytotoxicity and cell-cycle dysregulation, we used Western blotting to examine several kinases that have pro-survival activities or play roles in regulating the cell cycle. These experiments revealed that MT-6 induced a significant concentration- and time-dependent reduction in p-Akt and p-ERK levels, and increased p-JNK (Fig. 6A and B). Previous studies have reported that activation of JNK plays an important role in mitotic arrest, leading to inhibition of cancer development and apoptosis^31^,^32^. Cotreatment of cells with the JNK inhibitor, SP600125, significantly reversed MT-6–induced upregulation of mitotic arrest markers and Δψ_m_ loss (Fig. 6C and D), indicating that JNK plays a crucial role in MT-6–induced cell cycle dysfunction and Δψ_m_ loss. In addition, cotreatment with SP600125 also attenuated MT-6–induced cytotoxicity (Fig. 7A), accumulation of a subG1 population (Fig. 7B), and expression of apoptosis-associated proteins (Fig. 7C). Inactivation of JNK by siRNA also reversed MT-6-increased cell mitotic arrest and apoptosis (Supplementary Fig. S2). Moreover, cotreatment with SP600125 counteracted DR5 induction by MT-6 treatment, suggesting that MT-6 activates the JNK-DR5-apoptosis pathway.

**Figure 8:**
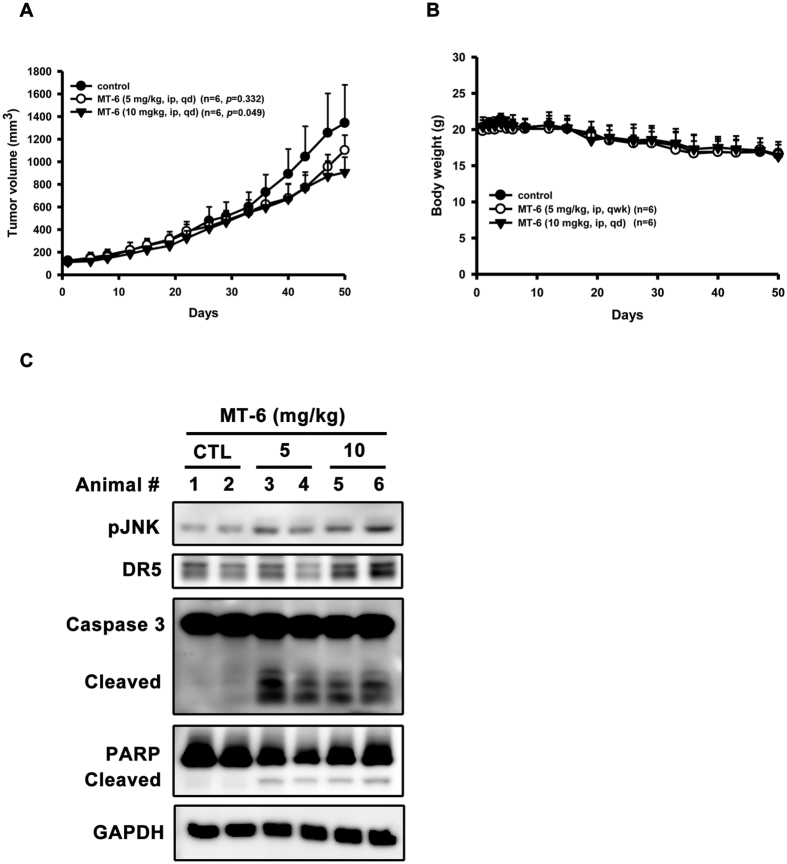
MT-6 inhibits tumor growth in SKOV3 xenografted mice. (**A**) MT-6 reduces tumor volume in an SKOV3 xenograft model. (**B**) MT-6 treatment did not cause significant loss of body weight in tested animals. Mice bearing established SKOV3 tumors (~100 mm^3^) were injected intraperitoneally with 5 or 10 mg/kg/d of MT-6 for 50 days, and tumor volumes were measured as described in Materials and Methods (n = 6 mice/group; *P < 0.05 compared with vehicle control). (**C**) Effects of MT-6 on intratumoral biomarkers in SKOV3 xenograft model. Tumors were harvested at terminal sacrifice and intratumoral proteins of two representatives from each group were collected and subjected to Western blot analysis.

To extend this analysis *in vivo*, we treated nude mice bearing established SKOV3 tumors with two different, low doses of MT-6. As shown in Fig. 8A, treatment with MT-6 at a dose of 5 or 10 mg/kg/d inhibited tumor growth by 19.6% and 35.5%, respectively, on day 50 post-treatment compared with vehicle controls. In addition, no adverse body weight loss was observed (Fig. 8B), suggesting that MT-6 treatment is relatively non-toxic. Moreover, intratumoral biomarkers in untreated or MT-6 treated tumors were also analyzed. The data revealed the MT-6 increased the phosphorylation of JNK and DR5 expression, and apoptosis as evidenced by the activation of caspase-3 and PARP (Fig. 8C).

## Discussion

Antimitotic agents have been used to treat various types of cancers and shown to provide significant therapeutic benefits for decades^3^,^8^,^33^, and efforts to develop novel mitotic inhibitors capable of overcoming drug resistance and exhibiting improved pharmacological profiles have continued^34^.

Although platinum-based combination chemotherapy (e.g., carboplatin plus paclitaxel) for patients with ovarian cancer has contributed to increased survival and higher responses compared with single agents, improvement in prognosis has still been limited by the intraperitoneal spread of malignancy^35^. Natural products have provided many lead structures for generating novel compounds with enhanced properties and have contributed significantly to cancer therapy over the past 30 years^36^. For instance, podophyllotoxin, the main constituent of *Podophyllum peltatum* (mayapple), is an effective treatment for various malignancies^37^. In addition to well-known cytotoxic drugs derived from plants, such as the Pacific yew *Taxus brevifolia* and *Catharanthus roseus*, numerous natural compounds have been found to exert multiple effects beyond their original applications^38^.

As we have shown here, MT-6 is a novel, potent antimitotic agent that induces apoptotic death in ovarian cancer cells. Previous reports have indicated the parent compound of MT-6, moscatilin, not only exhibits the ability to reduce fever as a traditional medicine, but also demonstrates significant antitumor activity in various cell types^20^. In the present study, we developed several lines of evidence suggesting that MT-6 acts via a different mechanism to disturb tubulin dynamics and shows more potent anti-proliferative activity. First, as shown in Supplementary Fig. S3, MT-6 promotes tubulin polymerization, as demonstrated by tubulin polymerization assays and confocal microscopy, whereas moscatilin inhibits tubulin polymerization^20^. Second, MT-6 is capable of inducing significant cytotoxicity in SKOV3, OVCAR3 and A2780 ovarian cancer cells (Fig. 1B–D). Third, MT-6 did not show significant cytotoxicity and no appreciate mitotic arrest and apoptosis were observed in normal uroepithelial SV-HUC-1 cells (Fig. 1E, and Supplementary Fig. S4). These findings demonstrate that, by virtue of its improved selectivity, MT-6 could be a better therapeutic compound because of its reduced side effects. The kinase activity of the CDK1-cyclin B1 complex is pivotal for mitotic entry, and during metaphase-anaphase transition, the expression level of cyclin B1 is reduced by 26 S proteasome-mediated degradation^39^. Our flow cytometry data indicate that MT-6 induces G2/M phase arrest, and Western blotting experiments showed that it upregulates mitotic markers (p-histone H3, cyclin B1, and MPM2), further leading to activation of apoptotic signaling by promoting cleavage of caspases and PARP in ovarian cancer cells (Fig. 2 and Supplementary Fig. S1). In addition to being the power generator in cells, mitochondria also plays a critical role in mediating activation of caspase proteases by inducing MOMP to trigger apoptotic cascades^40^. Tight regulation of pro- and anti-apoptotic members of the Bcl-2 family proteins controls the integrity of the mitochondrial outer membrane^41^. For example, the anti-apoptotic protein Bcl-2 inhibits Bax- and Bak-mediated mitochondrial outer membrane integrity breakdown, whereas phosphorylation of Bcl-2 leads to activation of Bax and Bak, causing the release of cytotoxic proteins into the cytosol and triggering caspase activation^42^. Our data show that MT-6 triggered a time-dependent Δψ_m_ loss and phosphorylation of Bcl-2 (Fig. 4), suggesting that a mitochondria-related mechanism of action is involved in MT-6–induced apoptosis.

Various environmental and genotoxic stresses lead to activation of mitogen-activated protein kinase (MAPK)—the so-called stress-activated protein kinase cascade that comprises two major pathways, JNK and P38 MAPK—and these signals are integrated into diverse cellular responses ranging from induction of apoptosis to increased cell survival^43^,^44^. For example, JNK-deficient cells fail to release mitochondrial apoptotic proteins and show no subsequent apoptotic response to environmental stress, indicating that JNK plays an important role in apoptosis^31^,^32^. However, JNK has also been reported to suppress apoptosis in IL-3–dependent hematopoietic cells through phosphorylation of the Bcl-2 family member, BAD^45^. Furthermore, inhibition of JNK activation by JNK antisense oligonucleotides (JNKAS) exerts different outcomes in different cancer cell types, inhibiting the growth of certain p53-deficient, but not p53-positive, tumor cells^46^,^47^,^48^. Some reports have indicated that activation of JNK by antimitotic agents is mediated by ASK1 (apoptotic signal-regulating kinase 1) and is p53-dependent^49^,^50^,^51^. These results suggest the anti-apoptotic or pro-apoptotic functions of JNK may be related to p53 status. However, our results indicate that JNK can be activated by MT-6, and MT-6–induced apoptosis is JNK-dependent in p53-mutant SKOV3 or p53 wild-type A2780 cells as evidenced by JNK inhibitor and JNK-knockdown (Fig. 7 and Supplementary Fig. S2B). Further, it has been found that, in cells arrested in mitosis in response to paclitaxel, activated JNK is associated with phosphorylated Bcl-2 in mitochondria^52^. Our study showed that MT-6 induces activation of JNK in a time- and concentration-dependent manner, and that cotreatment with the JNK inhibitor SP600125 significantly attenuated MT-6–induced Δψ_m_ loss (Fig. 6D), indicating that JNK plays a pivotal role in drug-induced cell death through mitochondria-mediated signal transduction.

Because TRAIL and TRAIL receptors (TRAILRs) have been largely characterized and shown to be involved in the extrinsic death pathway^53^,^54^, recombinant TRAIL and agonistic antibodies against TRAILRs capable of promoting TRAIL-mediated apoptosis in tumor cells have been evaluated for their clinical potential in cancer therapy^55^,^56^. Studies have also suggested that TRAIL can synergize with currently used anticancer drugs in combination therapy regimens^57^,^58^. Although TRAIL-mediated cell death in tumor cells shows significant therapeutic value, some reports have revealed potential toxicity to human hepatocytes, thymocytes, prostate epithelial and neural cells, thus raising safety concerns that these adverse outcomes may be related to TRAIL-mediated damage^59^,^60^,^61^,^62^,^63^,^64^. A previous study has indicated that ectopic overexpression of DR5 leads to cell death by transducing apoptotic signals through clustering of death domains^65^. Notably, elevated DR5 expression on the cell surface does not cause apoptosis in normal hepatocytes^66^, suggesting a degree of safety and effectiveness that would make DR5-mediated cell death in tumor cells a promising strategy for cancer therapy. Our results show that inhibition of JNK by SP600125 attenuated MT-6–triggered DR5 induction and prevented subsequent apoptotic cell death. In conclusion, our data suggest that MT6 is an effective anticancer agent *in vitro* and *in vivo.* MT6 disturbs microtubule dynamics by promoting tubulin polymerization, leading to G2/M cell cycle arrest and JNK activation, which in turn upregulates DR5 expression on the cell surface and stimulates subsequent mitochondria-related apoptotic signaling pathways by promoting a change in Δψ_m_. These findings provide evidences MT-6 may serve as a promising therapeutic drug for anticancer treatment in the future.

## Additional Information

**How to cite this article:** Chen, M.-C. *et al*. The apoptotic mechanisms of MT-6, a mitotic arrest inducer, in human ovarian cancer cells. *Sci. Rep.* **7**, 46149; doi: 10.1038/srep46149 (2017).

**Publisher's note:** Springer Nature remains neutral with regard to jurisdictional claims in published maps and institutional affiliations.

## Supplementary Material

Supplementary Figures
