# Supplementary material for: The apoptotic mechanisms of MT-6, a mitotic arrest inducer, in human ovarian cancer cells
Source: Sci Rep. 2017 Apr 7;7:46149. doi: 10.1038/srep46149 (PMC5384015; doi:10.1038/srep46149)
Supplement: Supplementary Figures [file srep46149-s1.pdf]

Supplementary Figures

The apoptotic mechanisms of MT-6, a mitotic arrest inducer, in human ovarian cancer cells

Mei-Chuan Chen[a], Yi-Chiu Kuo[b], Chia-Ming Hsu[c], Yi-Lin Chen[c], Chien-Chang Shen[d], Che-Ming Teng[b], Shiow-Lin Pan[c,*]

[a]Ph.D. Program for the Clinical Drug Discovery from Botanical Herbs, College of Pharmacy, Taipei Medical University, Taipei, Taiwan

[b]Pharmacological Institute, College of Medicine, National Taiwan University, Taipei, Taiwan.

[c]Ph.D. Program for Cancer Biology and Drug Discovery, College of Medical Science and Technology, Taipei Medical University, Taipei, Taiwan

[d]National Research Institute of Chinese Medicine, Ministry of Health and Welfare, Taipei, Taiwan

***Corresponding author:**

Shiow-Lin Pan, The Ph.D program for Cancer Biology and Drug Discovery, College of Medical Science and Technology, Taipei Medical University, 250 Wuxing Street, Taipei 11031, Taiwan.

E-mail: slpan@tmu.edu.tw

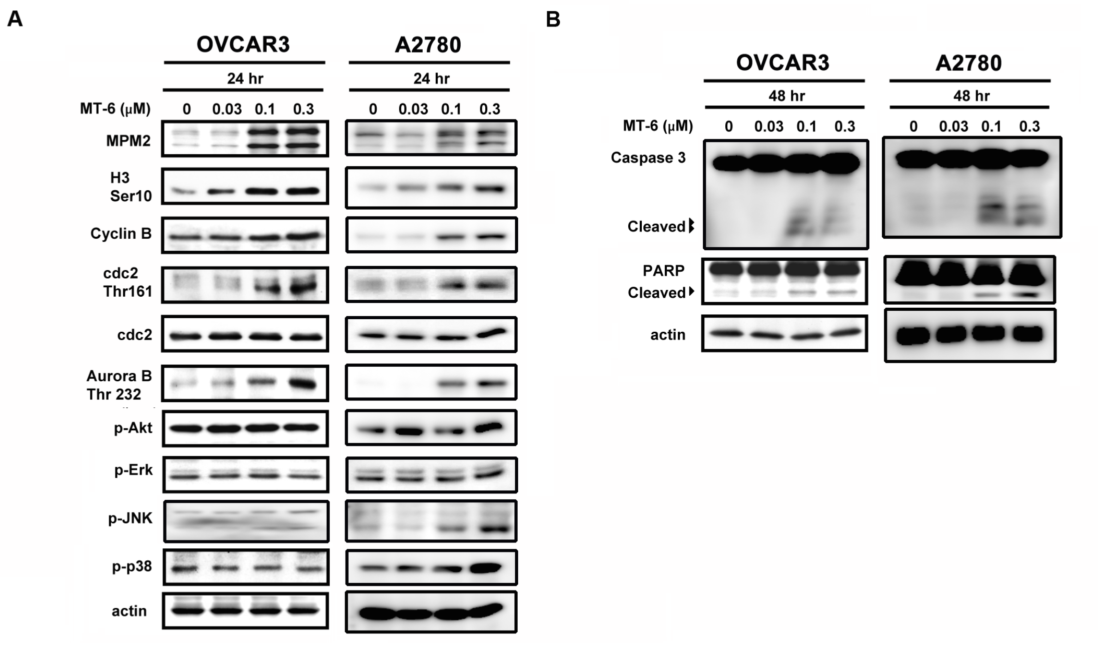

Supplementary Fig. S1. Effects of MT-6 on markers of mitotic arrest and apoptosis in OVCAR3 and A2780 cells. Cells were treated with the indicated concentrations of MT-6 for 24 h (A) or 48 h (B) and whole-cell extracts were analyzed by Western blotting.

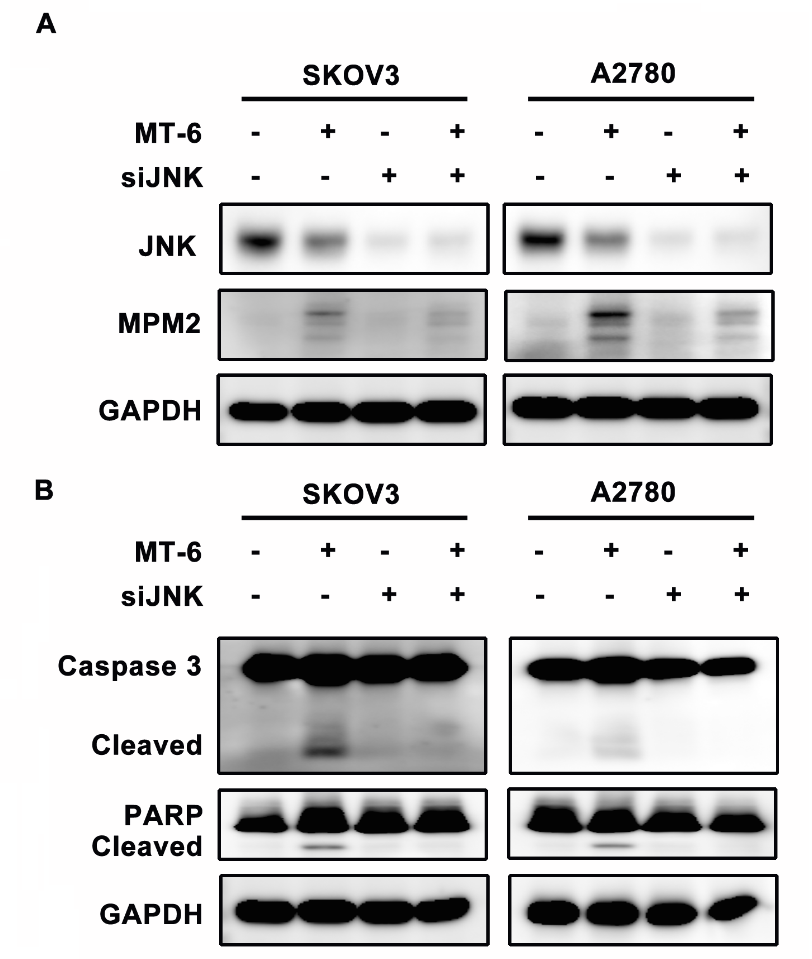

Supplementary Fig. S2. Effects of JNK-knockdown on MT-6-mediated mitotic arrest and apoptosis in OVCAR3 and A2780 cells. The cells were transiently transfected with siRNA against JNK for 24 h, and exposed to indicated concentrations of MT-6 for 24 h (A) or 48 h (B). Whole-cell extracts were subjected to Western blot analysis by indicated antibodies.

A

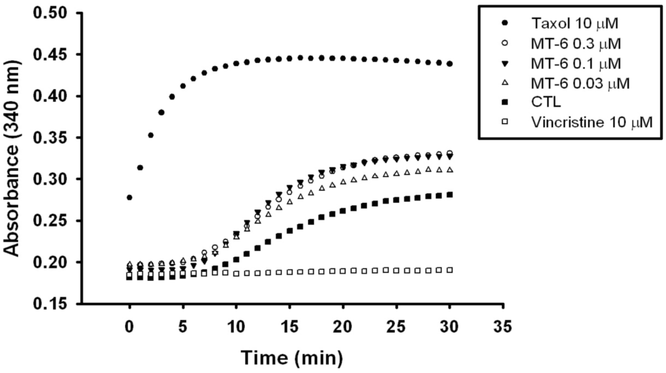

B

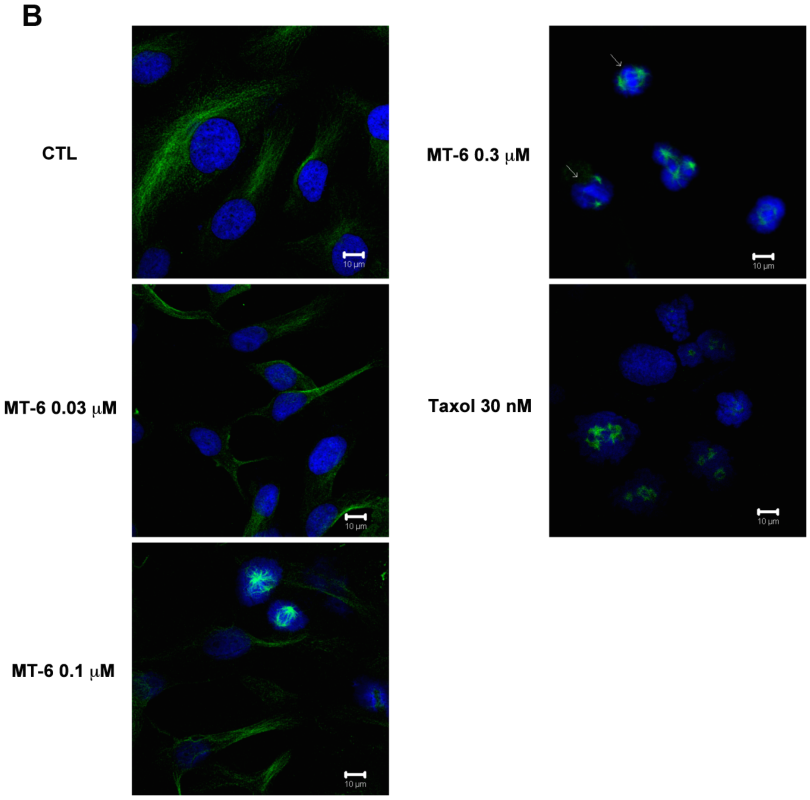

Supplementary Fig. S3. MT-6 induces tubulin polymerization and causes mitotic arrest in SKOV3 cells. (A) Tubulin in reaction buffer was incubated at 37°C in the presence of vehicle (DMSO), the indicated concentration of MT-6 (0.03–0.3 μM), 10 μM Taxol, or 10 μM vincristine. Effects on microtubule assembly were measured as described in Materials and Methods. (B) SKOV3 cells were incubated with DMSO, 0.03–0.3 μM MT-6, or 30 nM Taxol. The cellular microtubule network was analyzed by confocal microscopy using a monoclonal anti-β-tubulin antibody and FITC-conjugated mouse anti-mouse antibody, with DAPI counterstaining. Scale bar, 10 μm.

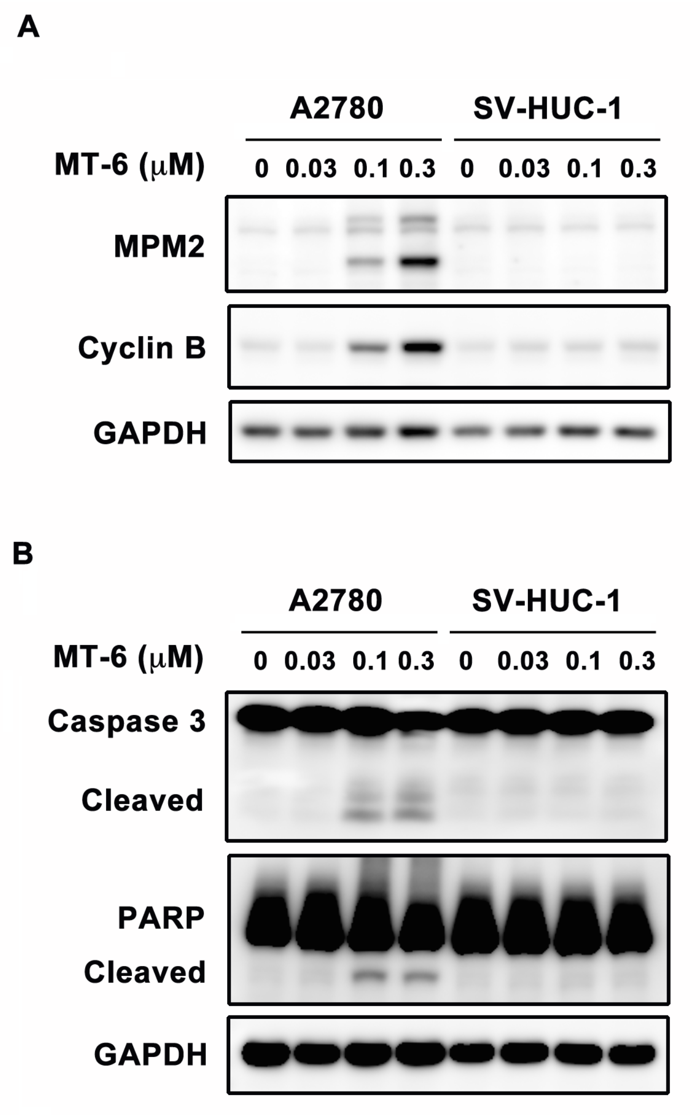

Supplementary Fig. S4. Comparison of MT-6 on mitotic arrest and apoptosis in cancer and non-cancer cells. A2780 and SV-HUC-1 cells were treated with MT-6 by indicated concentrations for 24 h (A) and 48 h (B). Total cell lysates were collected and subjected to Western blot analysis by indicated antibodies.
